# Dose–response association between Chinese visceral adiposity index and cardiovascular disease: a national prospective cohort study

**DOI:** 10.3389/fendo.2024.1284144

**Published:** 2024-04-18

**Authors:** Yongcheng Ren, Qing Hu, Zheng Li, Xiaofang Zhang, Lei Yang, Lingzhen Kong

**Affiliations:** ^1^ Henan Provincial Key Laboratory of Digital Medicine, Affiliated Central Hospital of Huanghuai University, Zhumadian, He’nan, China; ^2^ Institute of Health Data Management, Huanghuai University, Zhumadian, He’nan, China

**Keywords:** Chinese visceral adiposity index, cardiovascular disease, dose-response association, receiver operating characteristic, prospective cohort study

## Abstract

**Background:**

Chinese visceral adiposity index (CVAI) is a reliable visceral obesity index, but the association between CVAI and risk of cardiovascular disease (CVD) remains unclear. We explored the associations of CVAI with incident CVD, heart disease, and stroke and compared the predictive power of CVAI with other obesity indices based on a national cohort study.

**Methods:**

The present study included 7,439 participants aged ≥45 years from China Health and Retirement Longitudinal Study (CHARLS). Cox regression models were applied to estimate hazard ratios (HRs) and 95% confidence intervals (CIs). Restricted cubic splines analyses were adopted to model the dose–response associations. Receiver operator characteristic (ROC) analyses were used to compare the predictive ability of different obesity indices (CVAI, visceral adiposity index [VAI], a body shape index [ABSI], conicity index [CI], waist circumference [WC], and body mass index [BMI]).

**Results:**

During 7 years’ follow‐up, 1,326 incident CVD, 1,032 incident heart disease, and 399 stroke cases were identified. The HRs (95% CI) of CVD, heart disease, and stroke were 1.50 (1.25-1.79), 1.29 (1.05-1.57), and 2.45 (1.74-3.45) for quartile 4 versus quartile 1 in CVAI. Linear associations of CVAI with CVD, heart disease, and stroke were observed (*P*_nonlinear >_0.05) and per-standard deviation (SD) increase was associated with 17% (HR 1.17, 1.10-1.24), 12% (1.12, 1.04-1.20), and 31% (1.31, 1.18-1.46) increased risk, respectively. Per-SD increase in CVAI conferred higher risk in participants aged<60 years than those aged ≥60 years (*P*_interaction<_0.05). ROC analyses showed that CVAI had higher predictive value than other obesity indices (*P<*0.05).

**Conclusions:**

CVAI was linearly associated with risk of CVD, heart disease, and stroke and had best performance for predicting incident CVD. Our findings indicate CVAI as a reliable and applicable obesity index to identify higher risk of CVD.

## Introduction

Cardiovascular disease (CVD) is highly prevalent worldwide and its number nearly doubled from 271 million in 1990 to 523 million in 2019 (1, 2). The global trends for disability-adjusted life years and years of life lost also increased significantly and the number of CVD deaths increased steadily by 53.7% from 1990 to 2019 (2, 3). Among the modifiable risk factors for attributable CVD burden, high body mass index (BMI) ranked fifth and could worsen most other CVD risk factors (2, 4, 5). Unfortunately, 50.7% of Chinese adults were overweight or obesity in the most recent national survey (6), which may result in larger CVD burden. However, BMI might not fully capture cardiometabolic risk because of its failure in discriminating adequately between body fat mass and lean tissues or identifying regional body fat distribution (7, 8). Therefore, identifying reliable and applicable obesity indices would benefit to reducing CVD burden among Chinese population.

Recently, Chinese visceral adiposity index (CVAI), like visceral adiposity index (VAI) for Western population, was established to assess visceral adiposity among Chinese population and could predict metabolic disorders incidence well (9, 10). Numerous studies have demonstrated that CVAI has better performance than other obesity indices for predicting hypertension, diabetes, and their comorbidity (11–16). However, whether CVAI also predict incident CVD well among general population remains unclear. Studies based on rural population from a Chinese county or health examination population in a Beijing hospital reported positive associations between CVAI and stroke or coronary heart disease (17, 18), respectively. Another study from a Chinese province showed that CVAI was positively associated with CVD in women but not in men (19). Therefore, prospective cohort studies based on national data are needed to further validate CVAI as a visceral obesity index to predict incident CVD, which would provide additional epidemiological evidence for future CVD prevention.

Our study aimed to explore the dose–response associations of CVAI with CVD, heart disease, and stroke and compare the predictive ability of CVAI with that of other obesity indices (VAI, a body shape index [ABSI], conicity index [CI], waist circumference [WC], and BMI) based on China Health and Retirement Longitudinal Study (CHARLS).

## Methods

### Study population

The CHARLS, established in 2011, is an ongoing nationally representative cohort study focusing on adults aged ≥45 years in China. Details of the study design have been described elsewhere (20). In brief, a total of 17,708 participants were recruited from 150 counties of 28 provinces in China by a multistage probability sampling strategy from June 2011 to March 2012 and were followed up every-two years. The protocols of CHARLS were approved by the Biomedical Ethics Review Committee of Peking University and all participants provided signed informed consent before their enrollment.

The present study was conducted based on data from four waves (2011, 2013, 2015, and 2018) in the CHARLS. Of the 17,708 participants, we excluded participants with CVD or its unknown status at baseline (n= 3,118), CVD status at follow-up unknown (n= 1,015), younger than 45 years (n= 564), and missing data for age, BMI, WC, triglycerides (TG), and high-density lipoprotein cholesterol (HDL-C; n= 5,572). Finally, a total of 7,439 participants were included for the analysis.

### Data collection

The information about demographic characteristics, lifestyle factors, disease history was collected using standardized questionnaires by asking their age, gender, area, region, educational level, marital status, smoking, drinking, hypertension, diabetes, dyslipidemia, cardiovascular disease, cancer, liver disease, kidney disease, and medical history. Smoking was defined as smoking ≥100 cigarettes in their lifetime and drinking defined as drinking alcohol ≥12 times during the last year. BMI was calculated by dividing weight (kg) by the square of height. Hypertension was defined as systolic BP (SBP) ≥140 mm Hg and/or diastolic BP (DBP) ≥90 mm Hg and/or use of antihypertensive medication (21).

Blood samples were collected from participants after fasting overnight. Levels of fasting plasma glucose (FPG), total cholesterol (TC), TG, HDL-C, and low-density lipoprotein cholesterol (LDL-C) were measured by enzymatic colorimetric test, hemoglobin A1c measured by boronate affinity HPLC. Elevated TC was defined as TC ≥200 mg/dl. Diabetes was defined as fasting FPG ≥7.0 mmol/L, and/or non-fasting FPG ≥11.1 mmol/L, and/or hemoglobin A1c (HbA1c) ≥6.5%, and/or current treatment with anti-diabetes medication (22). The CVAI, VAI, ABSI, and CI were calculated as follows (9, 10, 23, 24):


$$\begin{array}{c} \text{CVAI (men) }=-267.93\;+\;0.68\;*\text{ age }+\;0.03\;*\text{ BMI }\\ \;+\;4.00\;*\text{ WC }\\ \;+\;22.00\;*\text{ Log}10\text{TG }-\;16.32\;*\text{ HDL-C} \end{array}$$

$$\begin{array}{c} \text{CVAI }\left(\text{women}\right)\;=-187.32\;+\;1.71\;*\text{ age }+\;4.23\;*\text{ BMI }\\ \;+\;1.12\;*\text{ WC }+\;39.76\;*\text{ Log}10\text{TG}\\ \;-11.66\;*\text{ HDL}-\text{C} \end{array}$$

$$\begin{array}{c} \text{VAI }\left(\text{men}\right)\;=\;(\text{WC}/\left(39.68\;+\;\left(1.88\;*\text{ BMI}\right)\right)\;*\;\left(\text{TG}/1.03\right)\\ \;\;*\;\left(1.31/\text{HDL-C}\right) \end{array}$$

$$\begin{array}{c} \text{VAI }\left(\text{women}\right)\;=\;(\text{WC}/\left(36.58\;+\;\left(1.89\;*\text{ BMI}\right)\right)\;*\;\left(\text{TG}/0.81\right)\\ \;\;*\;\left(1.52/\text{HDL}-\text{C}\right) \end{array}$$

$$\text{ABSI}=\text{WC}/\left({\text{BMI}}^{2/3}*{\text{height}}^{1/2}\right)$$

CI= WC/(0.109 × √weight/height)

### Outcome assessment

Accordant with previous studies (25, 26), incident CVD was assessed by the following standardized questions: “Have you been told by a doctor that you have been diagnosed with a heart attack, coronary heart disease, angina, congestive heart failure, or other heart problems?” or “Have you been told by a doctor that you have been diagnosed with a stroke?”. Participants who reported heart disease or stroke were defined as having CVD.

### Statistical analyses

Continuous data are described as median (interquartile range) and were analyzed by regression analysis to conduct trend tests among participants by quartiles of CVAI. Categorical data are presented as number (percentage) and were analyzed by Cochran-Armitage trend test.

Cox regression models were used to estimate hazard ratios (HRs) and 95% confidence intervals (CIs) for incident CVD, heart disease, and stroke by quartiles of CVAI. We also evaluated the effect of per standard deviation (SD) increase in CVAI through putting the value of CVAI divided by SD into the model. Model 1 was adjusted for age and gender; Model 2 was adjusted for model 1 plus area, region, educational level, marital status, smoking, and drinking; Model 3 was further adjusted for hypertension, diabetes, and TC. Dose–response associations between CVAI and incident CVD, heart disease, and stroke were assessed by restricted cubic splines (RCS) analysis incorporating four knots at the 5^th^, 35^th^, 65^th^, and 90^th^ percentiles, with the knot at 25^th^ percentile of the distribution as the reference. Subgroup analyses for per SD increase in CVAI across gender (men vs. women), age (<60 vs. ≥60 years), hypertension (no vs. yes), diabetes (no vs. yes), and elevated TC (no vs. yes) were conducted and its potential multiplicative interactions were tested. Sensitivity analyses were adapted by excluding participants with cancer, liver, and kidney disease at baseline.

The area under the receiver operating characteristic (ROC) curve (AUC) was used to evaluate the predictive value of CVD, heart disease, and stroke with CVAI, VAI, ABSI, CI, WC, and BMI. The differences among those AUCs were tested with the Z statistic. The RCS curves were modeled by R V.4.2.2, AUCs calculated by Medcalc v9.3, and other analyses involved using SAS V.9.4 (SAS Inst., Cary, NC). The Two-sided *P*< 0.05 was considered statistically significant.

## Results

### Baseline characteristics of study participants

Of the 7,439 participants included, the median age was 58.13 (13.16) years and 52.74% were women. The baseline characteristics for quartiles of CVAI are presented in **Table 1**. Participants in higher CVAI quartiles tended to be older, women, northerner, with hypertension, with diabetes, and have higher levels of TG, TC, VAI, ABSI, CI, WC, and BMI (*P*_trend<_0.05). The proportions of participants living in rural areas, marriage, smoking, drinking, and level of HDL-C decreased with increasing CVAI quartiles (*P*_trend<_0.05).

**Table 1:** Baseline characteristics of study participants.

	Quartile 1 (<67.41)	Quartile 2 (67.41-93.35)	Quartile 3 (93.35-122.67)	Quartile 4 (≥122.67)	*P*_trend_
Age (years)	56.16 (13.09)	57.26 (12.27)	58.53 (13.38)	60.77 (13.35)	<.0001
Women (%)	717 (38.57)	1,042 (55.96)	1,150 (61.93)	1,014 (54.49)	<.0001
Rural (%)	1,399 (75.26)	1,325 (71.16)	1,183 (63.70)	1,061 (57.01)	<.0001
Northern (%)	613 (32.97)	751 (40.33)	777 (41.84)	964 (51.80)	<.0001
Marriage (%)	1,672 (89.94)	1,658 (89.04)	1,637 (88.15)	1,595 (85.71)	<.0001
Higher school (%)	172 (9.25)	162 (8.70)	186 (10.02)	199 (10.69)	0.0657
Smoking (%)	842 (45.37)	554 (29.80)	444 (23.96)	476 (25.67)	<.0001
Drinking (%)	794 (42.71)	614 (32.98)	532 (28.65)	593 (31.86)	<.0001
TG (mg/dl)	74.34 (39.82)	92.93 (55.75)	115.93 (74.34)	154.88 (116.82)	<.0001
TC (mg/dl)	184.02 (44.46)	188.66 (47.55)	192.91 (47.94)	197.55 (51.42)	<.0001
HDL-C (mg/dl)	59.54 (20.49)	52.96 (17.40)	47.94 (15.85)	40.98 (13.92)	<.0001
HTN (%)	405 (21.79)	554 (29.75)	771 (41.52)	1,106 (59.43)	<.0001
DM (%)	139 (7.48)	185 (9.94)	274 (14.75)	438 (23.54)	<.0001
VAI	0.78 (0.64)	1.23 (1.00)	1.85 (1.62)	2.80 (3.03)	<.0001
ABSI	0.08 (0.01)	0.08 (0.01)	0.08 (0.01)	0.09 (0.01)	<.0001
CI	0.63 (0.05)	0.68 (0.04)	0.73 (0.05)	0.80 (0.06)	<.0001
WC (cm)	75.00 (6.60)	81.60 (5.50)	87.80 (5.70)	96.10 (7.90)	<.0001
BMI (kg/m^2^)	20.07 (2.65)	22.17 (2.88)	24.08 (3.16)	26.85 (3.91)	<.0001

Data are median (interquartile range) or number (%).

TG, triglycerides; TC, total cholesterol; HDL-C, high-density lipoprotein cholesterol; HTN, hypertension; DM, diabetes mellitus; CVAI, Chinese visceral adiposity index; VAI, visceral adiposity index; ABSI, A body shape index; CI, conicity index; WC, waist circumference; BMI, body mass index.

### Association of CVAI with risk of cardiovascular disease

**Figure 1:**
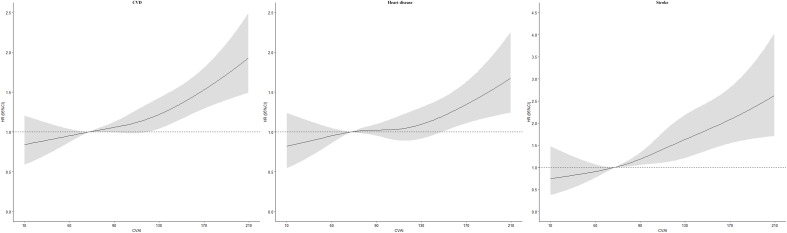
Dose–response association between Chinese visceral adiposity index and risk of cardiovascular disease HR, hazard ratio; CI, confidence interval; CVAI, Chinese visceral adiposity index; CVD, cardiovascular disease. Adjusted for age, gender, area, region, educational level, marital status, smoking, drinking, hypertension, diabetes, and total cholesterol.

During 7-year’ follow-up, a total of 1,326 incident CVD, 1,032 incident heart disease, and 399 stroke cases were identified. **Figure 1** showed positive linear associations of CVAI with CVD (*P* _nonlinear_ =0.4617), heart disease (*P*_nonlinear_ =0.3872), and stroke (*P*_nonlinear_ =0.9046). Risk of CVD, heart disease, and stroke increased across quartiles of CVAI (*P*_trend<_0.05; **Table 2**). In model 3, the multivariable adjusted HRs (95% CIs) for quartiles 2, 3, and 4 were 1.20 (1.00-1.44), 1.32 (1.11-1.58), and 1.50 (1.25-1.79) for CVD, 1.13 (0.92-1.38), 1.23 (1.01-1.50), and 1.29 (1.05-1.57) for heart disease, 1.63 (1.14-2.33), 1.65 (1.16-2.36), and 2.45 (1.74-3.45) for stroke. For per-SD increase in CVAI, the risk increased 17% (HR 1.17, 1.10-1.24) for CVD, 12% (1.12, 1.04-1.20) for heart disease, and 31% (1.31, 1.18-1.46) for stroke. Results of sensitivity analyses by excluding participants with cancer, liver, and kidney disease did not alter (**Supplementary Table S1**).

**Table 2:** Risk for cardiovascular disease by quartiles of Chinese visceral adiposity index.

	Quartile 1 (<67.41)	Quartile 2 (67.41-93.35)	Quartile 3 (93.35-122.67)	Quartile 4 (≥122.67)	*P* value
CVD					
Cases	211	296	352	467	
Incidence rate*	18.52	26.3	32.5	44.28	
Model 1	1.00	1.33 (1.11-1.59)	1.58 (1.32-1.87)	2.07 (1.75-2.45)	<0.0001
Model 2	1.00	1.25 (1.04-1.49)	1.46 (1.23-1.74)	1.80 (1.52-2.14)	<0.0001
Model 3	1.00	1.20 (1.00-1.44)	1.32 (1.11-1.58)	1.50 (1.25-1.79)	<0.0001
Heart disease					
Cases	171	236	280	345	
Incidence rate*	14.86	20.6	25.2	31.62	
Model 1	1.00	1.26 (1.04-1.54)	1.48 (1.22-1.8)	1.80 (1.49-2.18)	<0.0001
Model 2	1.00	1.16 (0.95-1.42)	1.34 (1.10-1.63)	1.51 (1.24-1.83)	<0.0001
Model 3	1.00	1.13 (0.92-1.38)	1.23 (1.01-1.50)	1.29 (1.05-1.57)	0.0116
Stroke					
Cases	49	84	94	172	
Incidence rate*	4.12	7.05	8.05	14.92	
Model 1	1.00	1.74 (1.22-2.48)	1.95 (1.38-2.77)	3.37 (2.44-4.65)	<0.0001
Model 2	1.00	1.73 (1.21-2.47)	1.94 (1.37-2.77)	3.30 (2.37-4.60)	<0.0001
Model 3	1.00	1.63 (1.14-2.33)	1.65 (1.16-2.36)	2.45 (1.74-3.45)	<0.0001

Risk of cardiovascular disease were presented as hazard ratio (HR) and 95% confidence interval (95% CI).

CVD, cardiovascular disease.

*Per 1000 person-years.

Model 1: Adjusted for age and gender;

Model 2: Adjusted for model 1 plus area, region, educational level, marital status, smoking, and drinking;

Model 3: Adjusted for model 2 as well as hypertension, diabetes, and total cholesterol.

We further explored the associations of per SD increase in CVAI with CVD, heart disease, and stroke in different age (<60 years vs ≥60 years), gender (men vs. women), hypertension (no vs. yes), diabetes (no vs. yes), and elevated TC (no vs. yes) groups (**Table 3**). Subgroup analyses showed that the risk of CVD, heart disease, and stroke were higher for participants aged<60 years than those aged ≥60 years (*P*_interaction_<0.05), with HRs (95% CIs) of 1.26 (1.16-1.37) and 1.11 (1.02-1.21) for CVD, 1.17 (1.06-1.29) and 1.10 (1.00-1.21) for heart disease, 1.51 (1.30-1.77) and 1.17 (1.02-1.35) for stroke, respectively. When stratified by gender, hypertension, diabetes, and elevated TC, multiplicative interactions were not observed for CVD, heart disease, and stroke (*P*_interaction _>0.05). Sensitivity analyses showed similar results (**Supplementary Table S2**).

**Table 3:** Association between per SD increase in Chinese visceral adiposity index and cardiovascular disease.

	CVD		Heart disease		Stroke	
	HR (95% CI)	*P* ^*^	HR (95% CI)	*P* ^*^	HR (95% CI)	*P* ^*^
**Total**	1.17 (1.10-1.24)		1.12 (1.04-1.20)		1.31 (1.18-1.46)	
**Age**		0.0010		0.0182		0.0044
<60 years	1.26 (1.16-1.37)		1.17 (1.06-1.29)		1.51 (1.30-1.77)	
≥60 years	1.11 (1.02-1.21)		1.10 (1.00-1.21)		1.17 (1.02-1.35)	
**Gender**		0.0589		0.2239		0.3204
Men	1.24 (1.15-1.35)		1.19 (1.08-1.31)		1.37 (1.20-1.56)	
Women	1.11 (1.02-1.22)		1.09 (0.98-1.20)		1.24 (1.04-1.48)	
**Hypertension**		0.1785		0.3760		0.0520
No	1.22 (1.11-1.33)		1.14 (1.03-1.26)		1.53 (1.30-1.80)	
Yes	1.12 (1.03-1.22)		1.09 (0.99-1.20)		1.18 (1.03-1.34)	
**Diabetes**		0.2612		0.2140		0.7580
No	1.15 (1.08-1.23)		1.10 (1.02-1.18)		1.32 (1.17-1.48)	
Yes	1.23 (1.07-1.42)		1.20 (1.01-1.42)		1.30 (1.04-1.63)	
**Elevated TC**		0.2917		0.6680		0.0900
No	1.19 (1.10-1.29)		1.12 (1.02-1.23)		1.41 (1.23-1.61)	
Yes	1.15 (1.05-1.26)		1.12 (1.01-1.25)		1.22 (1.04-1.43)	

SD, standard deviation, CVD, cardiovascular disease, HR, hazard ratio; CI, confidence interval; TC, total cholesterol.

^*^ Test for multiplicative interaction between subgroups.

Adjusted for age, gender, area, region, educational level, marital status, smoking, drinking, hypertension, diabetes, and total cholesterol.

### Predictive ability of CVAI and other obesity indices with cardiovascular disease

**Figure 2:**
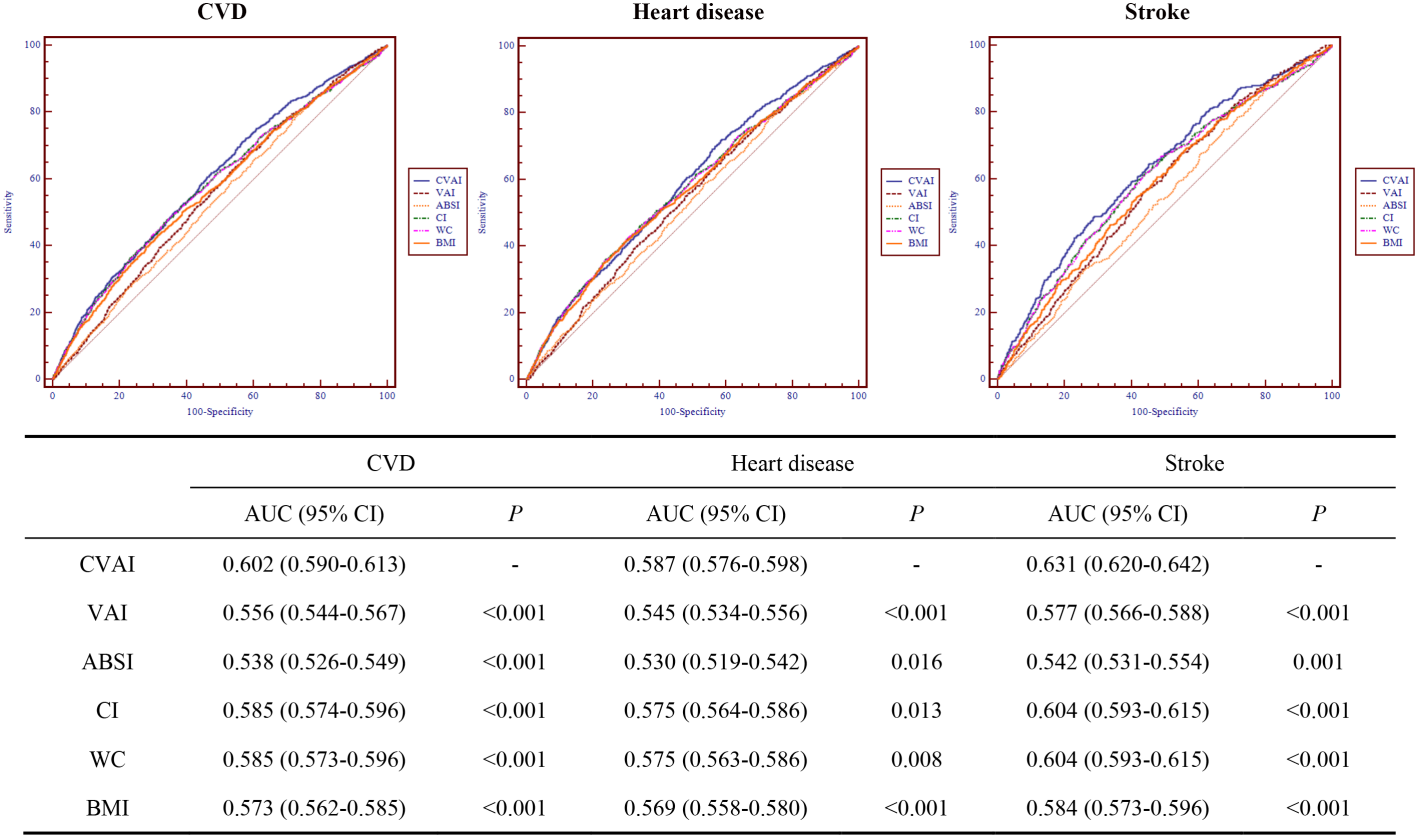
Receiver operating characteristic curves for CVAI, VAI, ABSI, CI, WC, and BMI for predicting cardiovascular disease. CVD, cardiovascular disease; CVAI, Chinese visceral adiposity index; VAI, visceral adiposity index; ABSI, A body shape index; CI, conicity index; WC, waist circumference; BMI, body mass index; AUC, area under the receiver operating characteristic curve; 95% CI, 95% confidence interval.

The ROC curves and AUCs for CVAI, VAI, ABSI, CI, WC, and BMI for predicting CVD, heart disease, and stroke is shown in **Figure 2**. The AUCs (95% CIs) for CVAI, were 0.602 (0.590-0.613) for CVD, 0.587 (0.576-0.598) for heart disease, 0.631 (0.620-0.642) for stroke, respectively. Among these obesity indices, CVAI had the largest AUC value for CVD, heart disease, and stroke, and the differences were all statistically significant (*P*< 0.05).

## Discussion

This prospective cohort study found linear associations of CVAI with CVD, heart disease, and stroke. Compared with quartile 1 of CVAI, the risk of CVD, heart disease, and stroke in quartile 4 increased 50%, 29%, and 145%, respectively. Per-SD increase in CVAI was associated with 17%, 12%, and 31% increased risk of CVD, heart disease, and stroke and confer higher risk in participants aged<60 years than those aged ≥60 years. Finally, we compared the ability of CVAI, VAI, ABSI, CI, WC, and BMI in predicting CVD, heart disease, and stroke and found CVAI as the best predictor among these obesity indices. Our study suggested CVAI as a reliable visceral obesity index for predicting CVD in Chinese adults.

Recent national survey showed that more than half of Chinese adults had overweight or obesity (6). Mounting evidence supported that visceral obesity was more strongly associated with CVD than overall obesity (27–29). Considering higher visceral fat in Asian people than Western people at a given BMI level (30, 31), evaluating visceral obesity would be more helpful for assessing risk of CVD among Chinese people. However, routine access to CT and MRI to assess visceral fat might be not suitable in general clinical practice and large cohort studies; thus, CVAI, constructed based on visceral fat by CT, was developed to evaluate visceral obesity among Chinese population (9). A study from a province in Southwest China study showed CVAI was associated with CVD in women but not in men (19). In our study, we visualized the association of CVAI with CVD and found positive associations in both men and women. Consistent with our findings, Zhao et al. found CVAI associated with stroke based on rural population of a Chinese county (17). We additionally found linear association between CVAI and stroke using national data, which was in line with Zhang et al. using logistic model (32). Similar to our findings, a study based on examination population in a Beijing hospital also found positive associations between CVAI and coronary heart disease in both men and women (18). Previous studies also reported that CVAI was positively associated with carotid atherosclerosis or carotid plaque (33, 34), which verified the rationality of the association between CVAI and CVD. Besides, we further explored the interaction of age, gender, hypertension, diabetes, and elevated TC on the associations of CVAI with CVD, heart disease, and stroke. Subgroup analyses showed participants aged<60 years had higher risk of CVD, heart disease, and stroke than those aged ≥60 years, which may be explained by longer duration of exposure to excess visceral fat.

Our results indicated that CVAI had the best ability to predict incident CVD, heart disease, and stroke among these obesity indices (CVAI, VAI, ABSI, CI, WC, and BMI). Previous studies also showed that CVAI was a better obesity indicator for coronary heart disease incidence than VAI, WC, and BMI and for stroke than other insulin resistance indices (17, 18). Besides, CVAI also had larger predictive value for cardiovascular events than VAI, WC, and BMI in Chinese diabetic patients (35, 36). A potential explanation for those findings may be that CVAI could evaluate visceral fat better in Chinese population (9). Contrast with measurement of visceral fat by CT and MRI (29, 37), CVAI, based on age, BMI, WC, TG, and HDL-C, are available and reliable to assess obesity in routine clinical practice and large cohort studies. Identifying participants with higher risk of CVD and then implementing lifestyle interventions may benefit to lower CVD risk.

Several potential mechanisms were suggested to explain the association between visceral obesity and CVD. Visceral adiposity promotes systemic and vascular inflammation by increasing production of interleukin-6, tumor necrosis factor-α, and high-sensitivity C-reactive protein, which lead to atherogenesis and subsequently incident CVD (38–40). Besides, visceral adiposity disturbs the renin–angiotensinogen system and produces excessive reactive oxygen species and reactive nitrogen species, and then induce oxidative stress, which could be presented as oxidized low-density lipoprotein, 8-hydroxylated deoxyguanosine, malondialdehyde, thioredoxin, and advanced oxidation protein products; the oxidative stress would induce a vicious cycle of endothelial dysfunction, inflammation, fibroblast proliferation and affect cerebral arteries through stenosis and occlusion, leading to CVD incidence (38, 41–44). Also, visceral obesity causes insulin resistance through increasing the production of inflammatory markers and adipocytokines and reducing that of adiponectin, which result in CVD incidence (45–47).

The present study comprehensively explored the associations of CVAI with CVD, heart disease, and stroke and compared the predictive ability of CVAI with other obesity indices (VAI, ABSI, CI, WC, and BMI) based on a national prospective cohort study. The findings are reliable and can be generalized to Chinese or other Asian people. Some limitations should be noted. First, we did not evaluate the effect of dynamic change in CVAI on CVD. Second, we failed to adjust for residual confounders, such as diet, air pollution, etc. Third, our study did not enable conclusions causal despite the prospective cohort design.

## Conclusions

Our study showed that CVAI was linearly associated with risk of CVD, heart disease, and stroke and CVAI had the best predictive ability among these obesity indices. The findings support CVAI as a suitable visceral obesity index for predicting CVD in Chinese or other Asian adults because of the availability of its components in large prospective studies and routine clinical practice.

## Data availability statement

The datasets presented in this study can be found in online repositories. The names of the repository/repositories and accession number(s) can be found below: This analysis uses data or information from the Harmonized CHARLS dataset and Codebook, Version D as of June 2021 developed by the Gateway to Global Aging Data. The development of the Harmonized CHARLS was funded by the National Institute on Aging (R01 AG030153, RC2 AG036619, R03 AG043052). For more information, please refer to https://g2aging.org/.

## Ethics statement

The studies involving humans were approved by public databases-China Health and Retirement Longitudinal Study (CHARLS). Besides, the protocols of CHARLS were approved by the Biomedical Ethics Review Committee of Peking University (IRB00001052-11015). The studies were conducted in accordance with the local legislation and institutional requirements. The participants provided their written informed consent to participate in this study.

## Author contributions

YR: Conceptualization, Data curation, Formal analysis, Funding acquisition, Project administration, Resources, Writing – original draft, Writing – review & editing. QH: Data curation, Writing – review & editing. ZL: Data curation, Investigation, Writing – review & editing. XZ: Data curation, Methodology, Writing – review & editing. LY: Conceptualization, Resources, Writing – review & editing. LK: Resources, Writing – review & editing.
