# Supplementary material for: Dose–response association between Chinese visceral adiposity index and cardiovascular disease: a national prospective cohort study
Source: Front Endocrinol (Lausanne). 2024 Apr 18;15:1284144. doi: 10.3389/fendo.2024.1284144 (PMC11063397; doi:10.3389/fendo.2024.1284144)
Supplement: Supplementary file 1 [file Table_1.docx]

**Table S1.** Sensitivity analysis for quartiles of Chinese visceral adiposity index by excluding participants with cancer, liver, and kidney disease.

|  | **CVD** | **Heart disease** | **Stroke** |
| --- | --- | --- | --- |
| **Quartile 1** | 1.00 | 1.00 | 1.00 |
| **Quartile 2** | 1.23 (1.02-1.49) | 1.17 (0.94-1.45) | 1.58 (1.09-2.29) |
| **Quartile 3** | 1.34 (1.11-1.62) | 1.25 (1.01-1.54) | 1.62 (1.12-2.34) |
| **Quartile 4** | 1.56 (1.29-1.89) | 1.34 (1.08-1.67) | 2.47 (1.74-3.53) |
| ***P* value** | <0.0001 | 0.0078 | <0.0001 |

CVD, cardiovascular disease.

Adjusted for age, gender, area, region, educational level, marital status, smoking, drinking, hypertension, diabetes, and total cholesterol.

**Table S2.** Sensitivity analysis for per SD of Chinese visceral adiposity index by excluding participants with cancer, liver, and kidney disease.

|  | **CVD** | **Heart disease** | **Stroke** |
| --- | --- | --- | --- |
| **Total** | 1.18 (1.11-1.26) | 1.13 (1.05-1.21) | 1.34 (1.20-1.49) |
| **Gender** |  |  |  |
| Men | 1.24 (1.13-1.35) | 1.16 (1.05-1.29) | 1.38 (1.21-1.58) |
| Women | 1.15 (1.05-1.27) | 1.12 (1.01-1.25) | 1.26 (1.05-1.52) |
| **Age** |  |  |  |
| <60 years | 1.29 (1.18-1.42) | 1.20 (1.08-1.34) | 1.56 (1.32-1.83) |
| ≥60 years | 1.12 (1.02-1.22) | 1.09 (0.99-1.21) | 1.18 (1.02-1.36) |
| **Hypertension** |  |  |  |
| No | 1.23 (1.13-1.35) | 1.16 (1.04-1.28) | 1.54 (1.31-1.82) |
| Yes | 1.13 (1.04-1.23) | 1.09 (0.98-1.21) | 1.21 (1.05-1.39) |
| **Diabetes** |  |  |  |
| No | 1.17 (1.09-1.26) | 1.11 (1.03-1.21) | 1.34 (1.19-1.51) |
| Yes | 1.22 (1.04-1.42) | 1.18 (0.98-1.41) | 1.33 (1.05-1.69) |
| **Elevated TC** |  |  |  |
| No | 1.20 (1.11-1.31) | 1.12 (1.02-1.23) | 1.44 (1.25-1.67) |
| Yes | 1.17 (1.06-1.29) | 1.15 (1.02-1.28) | 1.24 (1.05-1.45) |

SD, standard deviation; CVD, cardiovascular disease; TC, total cholesterol.

Adjusted for age, gender, area, region, educational level, marital status, smoking, drinking, hypertension, diabetes, and total cholesterol.
